# Pharmaceutical Peptides: From Synthesis and Mechanistic Pharmacology to Future Biologic Therapeutics

**DOI:** 10.3390/ph19060811

**Published:** 2026-05-22

**Authors:** Muhammad Yaseen Khan, Touseef Nawaz, Muhammad Sajid Hamid Akash, Adnan Amin

**Affiliations:** 1 Department of Pharmacy, Qurtuba University of Science and Information Technology, Peshawar 25100, Pakistan; 2 Department of Pharmaceutical Chemistry, Government College University, Faisalabad 54000, Pakistan; 3 Department of Life Sciences, Yeungnam University, Gyeongsan 38541, Republic of Korea

**Keywords:** peptide therapeutics, solid-phase peptide synthesis (SPPS), structural engineering, nanoformulations, targeted drug delivery, peptide–drug conjugates (PDCs), in silico drug discovery

## Abstract

Peptide therapeutics have emerged as a versatile class of biomolecules bridging the gap between small-molecule drugs and large biologics. Advantages of such molecules include high target specificity, potent bioactivity and reduced off-target toxicity. Despite these, broader clinical translation remains constrained by inherent limitations like poor metabolic stability, rapid renal clearance, limited membrane permeability and scalable synthesis. This review aims to systematically integrate advances in peptide science across natural discovery, synthetic methodologies, structural engineering, and translational delivery systems, while identifying critical research gaps hindering clinical adoption. We highlight diverse natural sources of bioactive peptides, including plant- (lunasin), animal- (Val-Pro-Pro (VPP) and Ile-Pro-Pro (IPP)), microbial- (nisin and cyclosporine), marine- (dolastatins) and venom-derived (chlorotoxin and ω-conotoxin MVIIA (ziconotide)) agents. Advances in solid-phase peptide synthesis (SPPS), green chemistry, and catalytic strategies are discussed alongside emerging in silico approaches, including artificial intelligence-driven sequence design and molecular modeling. Structural modifications such as cyclization, hydrocarbon stapling, PEGylation, and lipidation are critically evaluated for their role in enhancing pharmacokinetic and pharmacodynamic properties. Furthermore, nanoformulation strategies, including self-assembling peptides and cell-penetrating systems, are examined for their potential to overcome biological barriers. Importantly, this review identifies key unresolved challenges, including the lack of predictive models for peptide delivery systems, safety concerns associated with long-term modifications, and limited in vivo validation of naturally derived peptides. Addressing these gaps through integrated computational and experimental approaches will be essential for advancing next-generation peptide therapeutics. Collectively, this work provides a comprehensive framework for the rational design and translation of peptide-based precision medicines.

## 1. Introduction

The development of peptide-based drugs started in 1921 soon after the isolation of insulin, leading to the first use of a synthetic peptide drug [1]. Since then, over 100 peptide-based drugs have obtained regulatory approval from drug regulatory agencies, including the U.S. Food and Drug Administration (FDA), the European Medicines Agency (EMA), and the Chinese National Medical Products Administration (NMPA) [2,3]. Additionally, there are approximately 150 peptides currently being tested in clinical trials, and another 400–600 peptide therapeutics that are still in the pre-clinical trial stage [4,5]. In addition to this, at least seven other antimicrobial peptides (AMPs) have received FDA approval. Most of the approved AMPs were used topically, but they have also been administered systemically to treat serious cases of bacterial infection [6,7]. Peptide drugs are made up of short sequences of amino acids connected by peptide bonds. Typically, they contain fewer than 50 amino acid residues per sequence and their average weight ranges between 500 and 5000 Daltons [8]. Researchers today use various forms of chemical modification techniques to control physical characteristics of peptides, such as net charge, hydrophobicity, conformational stability, amphiphilicity, and amino acid sequences, so that they can be used to overcome peptide shortcomings, increase pharmacokinetic parameters, and improve biological potency [9,10].

Peptides show extremely high levels of target specificity and high binding affinities (commonly in the nanomolar to picomolar ranges) [11]. They generally cause little or no toxic side effects when they bind to non-specific proteins (off-target toxicity) and show lower immunogenicity than larger protein biologic drugs [12]. The benefits of peptides come from their ability to take on specific, defined three-dimensional structures for molecular recognition of biological receptors. Additionally, since peptides are smaller than most proteins, they create fewer opportunities for an adaptive immune response [13,14]. Peptides also exhibit pharmacokinetic/biopharmaceutical disadvantages, including reduced effectiveness in living organisms. Among the main limitations are: (1) rapid degradation by proteolytic enzymes (peptidases) in the gastrointestinal tract, blood, and tissues, resulting in decreased concentrations of peptides at the site of action [15]; (2) very short plasma half-lives (usually less than 30 min) caused by increased renal clearance due to their small sizes [16]; (3) limited membrane permeability (hydrophilic, charged groups), preventing passive transbilayer movement through cell membranes and therefore limiting their availability after oral ingestion [17]; and (4) increased entropy and potential for unfolding/aggregation under physiological conditions because of their inherent conformational flexibility [18].

Despite rapid growth in peptide therapeutics, current discussions often treat peptide sources, synthesis, structural modification, pharmacological mechanisms, and delivery strategies as separate topics. This fragmented view limits a clear understanding of how peptide sequence architecture, structure–activity relationships, pharmacokinetic behavior, and delivery performance collectively determine translational success. Therefore, this review aims to provide a focused and critical synthesis of pharmaceutical peptides by using selected representative examples to link peptide source, molecular design, mechanism of action, stability engineering and delivery strategy with therapeutic applicability. Particular emphasis is placed on distinguishing peptides with only in vitro bioactivity from candidates with stronger mechanistic and translational potential.

To improve focus, in this review, we selected representative examples to critically examine how peptide source, sequence architecture, structural modification, pharmacological mechanism and delivery strategy collectively determine translational potential. Particular attention is given to structure–activity relationships, pharmacokinetic limitations, and design strategies that convert bioactive peptide sequences into clinically usable therapeutic candidates.

## 2. Natural Sources of Peptides

Plant-, animal-, microbial-, marine- and venom-derived peptides provide sequence diversity, constrained scaffolds, post-translational motif and pharmacologically active templates that can guide subsequent synthetic and engineering strategies [13]. However, source-derived bioactivity alone does not establish therapeutic effects. Translation requires sequence definition, reproducible synthesis, mechanistic validation, pharmacokinetic optimization, delivery-system compatibility and in vivo efficacy and safety assessment [19].

### 2.1. Plant-Derived Peptides

Plant proteins, such as seed storage proteins, enzymatic components, and defense peptides, provide plant-specific protein architectures that differ from those found in mammals [20,21]. Soybean (Glycinin/β-Conglycinin), pea, and lentil hydrolysates have been identified as rich resources for angiotensin-converting enzyme (ACE) inhibitory and cholesterol-lowering peptide sequences [22]. These soybean, pea, and lentil hydrolysate-derived sequences also exhibit significant anti-diabetic activities through their ability to inhibit α-amylase and α-glucosidase activity [23]. Antioxidant peptides produced from cereal grains, including wheat and rice, contain high levels of sulfur, which enable them to act as chelators of metals and antioxidants [24]. In rapeseed and sesame seeds, the oilseed-derived sequences show a strong anti-inflammatory profile as they are able to suppress nuclear factor kappa B (NF-κB) pathways [25].

### 2.2. Animal-Derived Peptides

Animal-derived peptides for the most part originate from products made from animals; meat, dairy/milk products, and/or waste products derived from fish/seafood [26]. It is also possible to utilize a sustainable source of insect biomass to generate these peptides [27]. Utilizing the large quantities of protein in the many different forms of waste from foods can assist in creating circular economy processes by converting food waste with no other uses into therapeutic applications [28]. Additionally, the enzymatically generated peptides created from Myofibrillar (actin/myosin) and Sarcoplasmic proteins found in animal tissue have high concentrations of hydrophobic leucine, valine (Leu, Val) and aromatic phenylalanine, tyrosine (Phe, Tyr) amino acids [29,30]. These characteristics enable rapid movement across cellular membranes, where they can act as inhibitors to ACE. Casein is approximately 80% of Whey (~20%), both provide excellent starting points to produce antihypertensive peptides (Val-Pro-Pro, Ile-Pro-Pro), free radical scavengers, and opioid peptides (Casomorphins) via proteolytic cleavage of the two using either Pepsin or Trypsin [31]. Bioavailability is higher than that derived from mammalian origin when collagen and muscle proteins are extracted from fish/seafood [32].

### 2.3. Microbial-Derived Peptides

Microorganisms use a wide variety of highly developed, non-ribosomal peptide synthetase (NRPS) and polyketide synthase (PKS) routes to synthesize non-proteinogenic amino acids [33]. In addition, microorganisms can also carry out complex macrocyclization reactions using these same enzymes. The ribosomally synthesized and post-translationally modified peptides (RiPPs), such as the pore-forming nisin from Lactococcus lactis, are narrow-spectrum antibiotic alternatives that are ribosomally synthesized and then post-translationally modified [34]. Soil bacteria (*Bacillus*, *Streptomyces*) produce many types of surfactant-rich cyclic lipopeptides (such as surfactin, iturin) containing both D-amino acids and fatty acid tail groups [35]. Some fungi have been found to produce a number of very promising therapeutics, including cyclosporine (a cyclic undecapeptide produced by *Tolypocladium inflatum*, which targets specific T-cells for immunosuppressive activity) and plectasin (an agent capable of disrupting methicillin-resistant *Staphylococcus aureus* MRSA and cell wall integrity) [36]. A large variety of cyanobacterial hybrids has been identified as well. While some of these compounds are toxic to the liver (e.g., microcystins), they may offer new opportunities in the area of cancer therapy through the selective inhibition of PP1/PP2A protein phosphatases [37]. Lactic acid bacteria (LAB) are also being used to process food matrices into bioavailable products, utilizing cell surface-associated proteolytic enzyme systems to release pleiotrophic peptides (such as ACE inhibitory, immunomodulatory peptides) which would otherwise remain unavailable due to resistance to hydrolysis in the human gastrointestinal (GI) tract.

### 2.4. Algal and Marine-Derived Peptides

Seaweeds (Laminaria, Undaria) produce ACE inhibitory peptides that work together with their own, naturally occurring, sulfated polysaccharide (Fucoidan) for antihypertensive effects [38]. The peptides derived from algae (Chlorella, Spirulina) are from a bio-reactor of such high density and sustainability (up to 65% dry weight) that they may be used as an alternative to traditional livestock-based protein production [39]. They modulate the pathways of renin, ACE and DPP-IV (dipeptidyl peptidase-IV) and up-regulate the endogenous Nrf2 (nuclear factor erythroid 2-related factor 2) antioxidant cascade [24]. Sedentary marine animals have extreme chemical defense systems. The Cyclic Depispeptides (Didemnins) produced by tunicates and the Dolastatins produced by sponges are extremely cytotoxic [40,41]. However, these compounds have been re-purposed as conjugate payloads in FDA-approved antibody–drug conjugates (ADCs) like Brentuximab vedotin [42].

### 2.5. Venom-Derived Peptides

**Figure 1:**
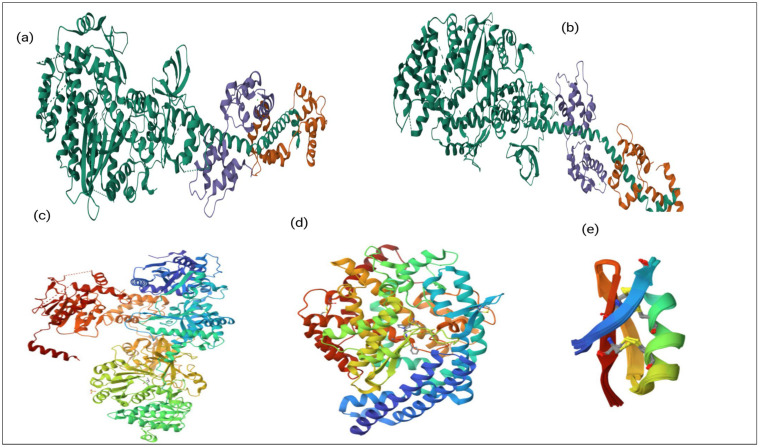
Three-dimensional structures of peptides from various sources, including plant- (Glycinin, 11S globulin, Protein Data Bank (PDB) Id 1FXZ) (**a**), animal- (Myoglobin PDB Id 1DFK) (**b**), microbial- (non-ribosomal peptide synthetase module, TycC, PDB Id 2VSQ) (**c**), algae- and marine- (ACE, target enzyme, human, PDB Id 1O86) (**d**), and venom-derived peptides (ICK peptide, ω-Agatoxin, PDB Id 1AGT) (**e**) Created and edited in BioRender. Zaman, W. (2026) https://BioRender.com/f66pzgm. Accessed on 20 April 2026.

Scorpion peptides such as chlorotoxin specifically bind to glioma cells, which enables them to inhibit voltage-gated ion channels [43]. They can be used as a tool for the early detection and treatment of cancer; they also serve as the first non-opioid pain killers [44]. Spider venom contains the inhibitor cystine knot (ICK) motif. ICKs have been proven to be resistant to proteolytic degradation and changes in pH. Therefore, ICKs make excellent scaffolds for rational design [45,46]. Snake venom has provided the structural template for the ACE inhibitors (captopril from Bothrops jararaca), and it has also produced disintegrins containing the RGD (arginine–glycine–aspartic acid) motif (eptifibatide for platelet aggregation) [47]. Cone snails produce conotoxins, which are heavily disulfide-bonded proteins that target specific calcium and sodium channels [48]. Synthetic ω-conotoxin MVIIA (Prialt/Ziconotide) (sequence:CKGKGAKCSRLMYDCCTGSCRSGKC-NH_2_), derived from cone snail venom, is approved by the FDA for use as an intrathecal analgesic [49]. Melittin from bee venom is a membrane lytic peptide that, at low doses, demonstrates potent anti-microbial and anti-inflammatory activity through inhibition of phospholipase A2 [50]. As with all other biological molecules, there are limitations associated with translating venom-based therapies. Off-target toxicities need to be reduced or eliminated; the complex process of correctly pairing the disulfides formed during in vitro synthesis needs to be overcome; and there is a risk of eliciting immune responses (Figure 1, Table 1).

Thus the source-based classification in this section provides the biological and structural starting point for the rest of the review. The following sections examine how these natural or source-inspired peptide motifs can be reproduced, diversified, and optimized through chemical synthesis, structural modification and delivery engineering.

## 3. Synthesis of Peptides and In Silico Discovery

### 3.1. Solid-Phase (SPPS) and Liquid-Phase (LPPS) Synthesis

Classical solution-phase peptide synthesis has been largely replaced by a new generation of solid-phase peptide synthesis (SPPS) [56]. A key feature of SPPS is that it allows the incorporation of each amino acid residue sequentially using a polymeric resin as a support for the growing peptide chain, with the C-terminus anchored to the resin [56]. This approach utilizes iterative cycles of coupling and deprotection, which can be achieved with high efficiency using either piperidine/Fmoc (9-fluorenylmethoxycarbonyl (Fmoc) or HF (hydrogen fluoride)/Boc (tert-butyloxycarbonyl) chemistry [57]. The use of highly reactive peptide coupling agents, such as DIC (N,N′-diisopropylcarbodiimide) or HATU (Hexafluorophosphate Azabenzotriazole Tetramethyl Uronium), in approximately 3–5-fold excess, provides a sufficient driving force to achieve near complete conversion of reactants [56,58]. Unreacted starting material may then be removed through washing. However, the longer a peptide is synthesized via SPPS, the higher the potential for premature termination during its synthesis due to the occurrence of deletions and/or truncations [59]. An alternative method, liquid-phase peptide synthesis (LPPS), addresses these issues by utilizing soluble “tags” (fluorous, PEG (polyethylene glycol), etc.) attached to the peptide during its synthesis [60]. These tags allow for the homogenous reaction kinetics that occur during LPPS, yet they also provide a means for isolating the pure product through simple precipitation of the tag out of organic solvents. Finally, for very long peptides, convergent native chemical ligation (NCL) provides a seamless way to join separately synthesized segments of a peptide together through a single-step transthioesterification reaction occurring at N-terminal cysteine residues on each segment [61,62].

### 3.2. Green-Chemistry Innovations

The conventional solid-phase peptide synthesis (SPPS) process creates an enormous volume of hazardous waste as it utilizes large amounts of reprotoxins such as DMF (N,N-dimethylformamide)/NMP (N-methyl-2-pyrrolidone) in the range of 1000 to 5000 L per kilogram of synthesized peptide [63]. The development of green-chemistry processes has greatly reduced the amount of hazardous materials used during the synthesis process, in that they have replaced traditional hazardous solvents with environmentally friendly, biodegradable solvents such as GVL9 γ-valerolactone, Cyrene, and TEP (triethyl phosphate) [64]. Simultaneously, Microwave-Assisted SPPS (MW-SPPS) dramatically reduces the time needed for each standard coupling reaction from hours to 2 to 10 min, resulting in a significant reduction in energy usage, which ranges from 30 to 60% [65]. Additionally, MW-SPPS minimizes unwanted thermal-side reactions. In order to increase atom economy, continuous-flow SPPS (CF-SPPS) operates reagents through bed reactors and continuously monitors the UV (ultraviolet) absorbance in real time while running at almost stoichiometric ratio levels [66]. The use of wash-free methodologies using recycled ionic liquids or solid-supported scavengers results in significantly lower volumes of total solvents being required.

A more fundamental green-chemistry strategy is to reduce or avoid protecting-group operations during peptide synthesis. Conventional peptide synthesis often requires repeated cycles of protection, deprotection, activation, coupling, and purification [67]. Therefore, methods that enable peptide-bond formation between unprotected or minimally protected amino acids can substantially reduce synthetic step count, reagent consumption, and waste generation [68]. Transient masking with silylating reagents has enabled peptide-bond formation from unprotected amino acids and peptides by temporarily controlling reactive amino and carboxyl groups during coupling [69]. Practical N-to-C peptide synthesis with minimal protecting groups has further demonstrated that peptide elongation can be achieved through catalytic peptide-thioacid formation and oxidative peptide-bond formation [70]. Recent inverse peptide synthesis strategies using transiently protected amino acids have expanded this concept by reversing the conventional direction of peptide-chain construction and enabling efficient one-pot transient protection, activation, aminolysis and in situ deprotection [71]. Most recently, convergent peptide-bond formation between unprotected amino acids has shown that oligopeptides can be assembled from minimally manipulated building blocks [72]. Complementary green-synthesis approaches also support this direction, including peptide-bond formation using unprotected N-carboxyanhydrides under liquid-assisted grinding conditions, side-chain-unprotected solid-phase peptide synthesis using greener solvent systems and tantalum-catalyzed peptide elongation of unprotected amino acids using N-trimethylsilylimidazole [73].

### 3.3. Display Technologies

The success of high-throughput peptide discovery requires an association of a peptide’s phenotypic properties with its genotypic attributes [74,75]. Phage display was the first method for achieving such physical linkage of phenotype and genotype through the expression of large libraries of 10^9^–10^10^ peptides on M13 viral coats [74,76]. Cell-free systems using mRNA and ribosome displays (such as the RaPID system), which use puromycin linkage to quickly screen 10^13^–10^14^ different peptide variants, including non-natural and macrocyclic amino acids, will overcome the limitations placed on the number of cells that can be transformed [77,78,79]. Importantly, the ability of mirror-image phage displays to directly address the problem of peptide fragility; by rapidly screening large numbers of standard L-peptide libraries against highly synthetic D-enantiomer targets, researchers may easily find sequences that are essentially completely resistant to degradation in vivo due to being “invisible” to all endogenously expressed protease enzymes and have exceptionally long in vivo half-lives [80,81].

### 3.4. Artificial Intelligence (AI) and Computational Modeling

**Figure 2:**
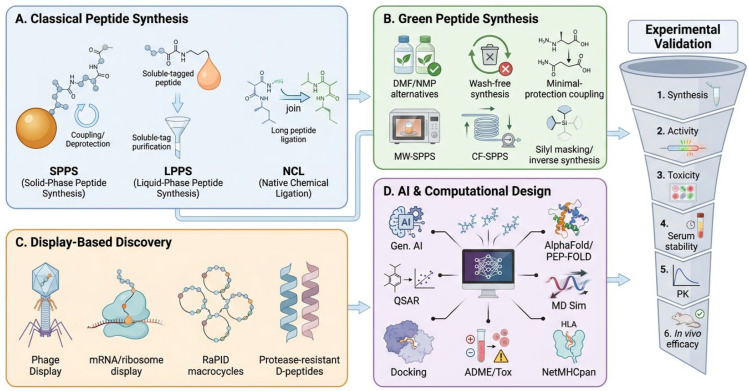
Overview of peptide synthesis and in silico discovery techniques. This figure provides a comprehensive overview of the key peptide synthesis methods and in silico discovery techniques. It integrates solid-phase peptide synthesis (SPPS), liquid-phase peptide synthesis (LPPS), and emerging catalytic techniques, as well as green-chemistry innovations. The figure also highlights advancements in display technologies and the integration of artificial intelligence (AI) and computational modeling in peptide discovery. Created and edited in BioRender. Zaman, W. (2026) https://BioRender.com/f66pzgm. Accessed on 20 April 2026.

AI is moving peptide discovery away from empirical screening toward rational design [82]. While generative deep learning architectures (LSTMs (long short-term memory networks), VAEs (variational autoencoders), GANs (generative adversarial networks)) generate entirely new bioactive sequence data, QSAR (Quantitative Structure–Activity Relationships) models utilize mathematical relationships between structural characteristics and biological activity [83,84]. In order to address the large number of conformations that are accessible to a peptide, predictive engines (AlphaFold, PEP-FOLD), and Molecular Dynamics (MD) simulations determine specific three-dimensional folding sets of an ensemble, as well as the pathways by which a peptide inserts into membranes [85]. The binding affinity of a peptide for a particular protein target is also predicted through the use of flexible molecular docking algorithms (Schrodinger Suite, Rosetta FlexPepDock, AutoDock Vina version 1.1.2) [86,87]. Prior to the actual chemical synthesis of these peptides, ADME/Tox prediction algorithms have been developed in silico to predict potential PK (pharmacokinetics) liability issues of each synthesized peptide; specifically predicting metabolic proteolytic cleavage sites, modeling renal excretion rates, and using tools such as NetMHCpan to identify T-cell epitopes that may be recognized by an immune system, thereby identifying potential targets for deimmunization [88] (Figure 2).

Several experimentally validated studies show that predictive peptide-design methods have progressed beyond theoretical screening and can now generate or prioritize candidates for synthesis and biological testing. In antimicrobial peptide discovery, AMPGAN v2 introduced a bidirectional conditional generative adversarial network for controlled AMP generation. Importantly, the earlier AMPGAN proof-of-concept promoted six top-ranked computational candidates to synthesis and antibacterial assays, of which three showed broad-spectrum antibacterial activity [89]. Deep learning-guided proteome mining has also produced experimentally validated candidates, as shown by APEX 1.1, which mined 233 archaeal proteomes and identified 12,623 putative antimicrobial molecules; 80 archaeasins were synthesized, 93% showed in vitro antimicrobial activity, and archaeasin-73 reduced *Acinetobacter baumannii* burden in mouse infection models with activity comparable to polymyxin B [90]. More recently, a ProteoGPT/AMPGenix pipeline combined protein-language-model-based generation, candidate filtering, toxicity assessment, and wet-lab validation. The aim of investigation was to identify generated and mined AMPs. The g_AMP14, g_AMP33, g_AMP42, m_AMP46, and m_AMP76 significantly reduced CRAB (arbapenem-resistant *Acinetobacter baumannii*) burdens in a mouse thigh infection model, whereas g_AMP14, g_AMP35, m_AMP46, and m_AMP76 reduced MRSA burdens and weaker in vivo performance of some peptides was linked to lower serum-protease stability [91]. Structure-guided computational design has also produced target-specific peptide-like/miniprotein binders, including SARS-CoV-2 spike-binding designs such as LCB1 and LCB3. These were generated by de novo computational design and experimentally validated as potent neutralizing candidates [92]. These examples indicate that AI and molecular modeling are most useful when coupled with synthesis, mechanistic assays, serum-stability testing, toxicity profiling, and in vivo validation, rather than being used as standalone prediction platforms.

## 4. Classification of Peptides Based on Structure

The structure that therapeutic peptides are created in determines both the pharmacokinetics of a peptide and its ability to specifically interact with the intended biological target. Native peptides exhibit inherently poor biopharmaceutical properties. Structural reinforcements for drug design include the backbone cyclization of a peptide; complex networks of disulfides (cystine-knot inhibitors); and hydrocarbon stapling to stabilize α-helix secondary structures within the peptide backbone and provide proteolytic stability [93]. Beyond geometric stabilization, many modifications occur at the sequence level to protect against enzymatic cleavage by incorporating non-native amino acids (D-amino acids and N-methylated amino acids) and through covalent conjugation of other macromolecules, including lipids, sugars, and polyethylene glycol (PEG) [94]. Overall, when these stabilized scaffolds are combined with cytotoxic moieties (peptide–drug conjugates) or with self-assembling amphipathic motifs, they can be converted from basic receptor agonists to complex supramolecular delivery vehicles [95] (Table 2).

## 5. Mechanistic Pharmacology of Peptide Therapeutics

The use of peptides as molecules that are capable of switching on/off large numbers of receptors has shown significant promise [103]. They have been demonstrated to be used as agonists/antagonists at G-protein coupled receptors (GPCRs), including GLP-1 (glucagon-like peptide-1) analogs for blood glucose lowering [103,104]; they interact with RTKs (receptor tyrosine kinases) to either stimulate or inhibit tissue repair/regeneration, as well as suppress or enhance oncogenesis [105]; they also bind to ion channels through their interactions with toxins derived from snake venoms, which allow for precise, opioid-free pain relief [49]. In addition, peptides have been utilized to competitively or allosterically block key steps within various enzymatic pathways [106]. Examples of potential applications of peptides include: cardiovascular disease treatment using ACE inhibitors and renin inhibitors, metabolic disorders treated with DPP-IV and alpha-glucosidase inhibitors, and the treatment of viruses and cancers using peptide-based proteolytic enzyme inhibitors [107].

Antimicrobial cationic peptides selectively target negatively charged lipid membranes found in bacteria compared to the zwitterionic lipid membrane of mammals [108]. These peptides elicit fatal lysis through one of three different architectures: detergent-like surface accumulation (carpet model); channel formation through cooperative interaction between the peptide and lipid (toroidal pore model); and purely peptidic trans-membrane channels (barrel-stave model) [108,109]. Modified and/or cell-penetrating peptides can now provide access to previously considered “undruggable” intracellular signal cascades [81]. Through the disruption of BCL-2 (B-cell lymphoma 2) family proteins, modified peptides can initiate programmed cell death in malignancies by inducing mitochondrial apoptosis [110]. Additionally, modified peptides may significantly reduce inflammation through the down-regulation of the NF-kappa B, MAPK (mitogen-activated protein kinase), and PI3-K (phosphoinositide 3-kinase)/AKT (protein kinase B) signaling axes [111].

ACE-inhibitory peptides provide a useful example of why therapeutic interpretation should be based on sequence-defined pharmacology, not only on peptide source. ACE is a zinc-dependent dipeptidyl carboxypeptidase that contributes to blood-pressure regulation by converting angiotensin I into the vasoconstrictor angiotensin II and by participating in bradykinin degradation [112]. Therefore, ACE inhibition may reduce angiotensin II-mediated vasoconstrictive signaling while supporting kinin-associated vasodilatory tone [113]. The activity of ACE-inhibitory peptides is strongly influenced by the C-terminal region of the sequence because ACE substrate recognition and inhibitor binding depend substantially on the COOH-terminal dipeptide/tripeptide architecture [114]. Structural evidence from the human ACE–lisinopril complex further shows that ACE inhibition involves accommodation within a defined catalytic pocket containing a zinc-binding environment and substrate-recognition subsites [112]. Mechanistically, ACE-inhibitory peptides may act through competitive or mixed-type inhibition involving hydrogen bonding, electrostatic interactions, hydrophobic packing, and spatial proximity to the catalytic Zn^2+^ environment [115]. Val-Pro-Pro and Ile-Pro-Pro represent comparatively stronger translational examples because clinical studies have reported improvements in central systolic blood pressure, arterial stiffness, and endothelial function after intake of casein hydrolysates containing these tripeptides [116]. Experimental studies further suggest that these peptides may improve vascular function not only through ACE inhibition but also through nitric oxide-associated endothelial relaxation and attenuation of arterial dysfunction in hypertensive animal mode [117].

Peptides’ ability to mitigate oxidative damage in cells is due to a triad of defenses: direct scavenging of reactive oxygen species by donating electrons from aromatic and sulfurous amino acid residues; binding of transition metals, preventing generation of toxic Fenton reaction products; and stimulation of endogenously produced antioxidant enzymes (heme oxygenase-1, NAD(P)H quinone oxidoreductase-1, glutathione biosynthetic enzymes) by activation of the transcription factor Nrf2 [118]. Modified peptides are able to regulate both innate and adaptive immunity. They can activate innate immunity (chemotaxis and TLRs); suppress autoimmune responses that lead to transplant rejection; and generate neoantigens that are recognized by the host immune system against tumor antigens, resulting in a highly specific therapeutic intervention.

## 6. Structural Engineering for Improved Pharmacokinetic Properties

### 6.1. Modulating Fundamental Physicochemical Factors

The use of a rational approach for designing therapeutic peptides is dependent upon the balance of net charge and hydrophobicity in order to determine how a peptide will partition within the cell membrane, enter into cells, and establish the therapeutic index between efficacy toward a desired target (i.e., antimicrobial action) and cytotoxicity to the host red blood cell [119]. A positive charge density in therapeutic peptides typically ranges from +2 to +9 and is responsible for the electrostatic attraction toward negatively charged targets [120]. Moderate hydrophobicity (typically 30–60% hydrophobic) allows for interaction with lipid bilayers [121]. Amphipathicity is achieved by positioning hydrophobic and hydrophilic amino acid side chains on opposing surfaces of an alpha helix or beta sheet, enabling therapeutic peptides to be precisely oriented at membranes where they either cause damage to the targeted cell [122].

In addition to general physicochemical characteristics of therapeutic peptides that influence their function, there are distinct structural activity relationships (SAR) governing each functional property based upon unique sequence motifs [123]. For example, in antioxidant peptides, the placement of certain aromatic (tryptophan, tyrosine, phenylalanine), as well as sulfur-containing residues, is critical [118]; ACE-inhibiting tri-peptide sequences require C-terminal hydrophobic residue placement [124] and penetration across cellular membranes requires arginine-rich clusters [125]. Therefore, using both predictive indexes such as GRAVY (grand average of hydropathy) and Boman, along with multi-dimensional SAR analysis, it is possible for researchers/developers to select those parameters that provide optimal interaction between therapeutic peptides and cellular targets [126].

Net charge is not only a determinant of peptide potency but also an important regulator of pharmacokinetics, biodistribution, and tolerability. Studies using radiolabeled peptide models show that even single-residue charge changes can significantly alter renal uptake, indicating that charge modulation may influence kidney retention and systemic clearance [127]. In cationic antimicrobial peptides (AMP), increased positive charge strengthens the interaction with negatively charged bacterial membranes, but excessive cationicity may also increase non-specific binding to mammalian membranes and reduce selectivity [128]. Similarly, highly cationic cell-penetrating peptides can improve cellular entry but may show rapid tissue distribution and liver accumulation after systemic administration, limiting stable plasma exposure [129]. In contrast, long-acting GLP-1 receptor agonists demonstrate that charge must be optimized together with hydrophobicity, linker design, enzymatic stability, and albumin binding to extend systemic exposure and improve dosing feasibility [130]. Thus, net charge should be balanced carefully because the same electrostatic features that enhance target interaction may also affect renal handling, tissue retention, plasma–protein association, and systemic safety.

### 6.2. Conformational Stabilization: Cyclization, Stapling, and Disulfide Bridges

Backbone cyclization in peptides (head-to-tail or side chain to backbone) provides enhanced stability by eliminating sites for exopeptidase hydrolysis and preorganizing the sequence into a rigid bioactive conformation [131]. By doing so, it greatly reduces the entropic penalty associated with binding to the receptor [132]. This structural lock is well illustrated by cyclitides from plants that contain both a circular backbone and a Cyclic cystine knot (CCK), which interlock to form an ultrastable platform capable of resistance to boiling water temperatures and gastric fluids [133,134]. To have access to intracellular targets that are usually thought to be “undruggable”, structural engineering utilizes peptide stapling [135]. Ruthenium catalyzes olefin metathesis in order to introduce hydrocarbon cross-links spanning either one or two helical turns; therefore, the peptide is stabilized into a very stable alpha helix structure through these hydrophobic staples [136]. The amount of increase in proteolytic resistance and passive trans cellular permeability, as well as an exponential increase in helicity (>80%), has been shown by a number of examples, including the p53-MDM2/MDMX antagonist, ALRN-6924 [137].

Stabilization of sulfur-containing motifs also enhances tertiary structures and prevents them from being metabolically degraded [138]. Native disulfide bridges provide a limited set of rigid geometries that determine the receptor specificity for a given protein [45,59,139]. However, thioether bridges that cannot be reduced can stabilize the motif even when it is located in a reducing environment such as the cytosol [140]. In addition, replacement of certain backbone carbonyls with thioamides (thiocarbonyls) provides subtle adjustments in hydrogen bonding geometry, which results in the modification of the peptide being essentially insensitive to serine and cysteine protease attack while retaining its native target complementarity [139].

### 6.3. Chemical Modifications for Extended Half-Life

To improve the bioavailability and increase the range of systemic circulation of peptides, they are chemically modified in order to surmount the limitations imposed by their pharmacokinetics [141]. The backbone is either N-methylated or else the chirality is altered (for example, D-amino acids or other non-standard amino acids), which greatly enhances trans-cellular permeability and renders the peptide resistant to degradation by endogenously produced proteolytic enzymes [142]. Additionally, macromolecular conjugates can be attached to the peptide to preclude its premature elimination via glomerular filtration with the expansion of the molecular volume; lipidation accomplishes this goal by providing an interaction site on albumin and forming a slowly releasing depot (e.g., GLP-1 agonist) [104], while PEGylation provides a steric ‘cloud’ about the molecule that both reduces filtration and reduces antibody-mediated immunity [143]. Glycosylation also serves multiple functions, including increasing aqueous solubility, masking immunogenic epitope sites from neutralizing antibodies, and facilitating targeted delivery to specific tissues [144].

Albumin-binding modification of GLP-1 receptor agonists provides a clear quantitative example of peptide pharmacokinetics transformation by chemical engineering. Native GLP-1 is rapidly degraded and cleared, with experimental pharmacokinetic data showing a plasma half-life of approximately 1.5–2.0 min, which explains why unmodified GLP-1 is unsuitable for intermittent therapeutic dosing [145]. Shorter-acting GLP-1 receptor agonists that do not rely on strong albumin-binding protraction, such as exenatide, show longer but still limited systemic exposure, with population pharmacokinetic analysis reporting an elimination half-life of approximately 2.4 h and predominantly renal elimination [146]. In contrast, liraglutide contains a fatty-acid modification that facilitates high plasma–protein binding; an in vitro protein-binding study showed that more than 98.9% of liraglutide was protein-bound at clinically relevant concentrations, providing a mechanistic basis for delayed clearance and once-daily administration [147]. Consistent with this protraction strategy, early clinical pharmacokinetic studies of NN2211/liraglutide reported a subcutaneous half-life of approximately 11–15 h [148]. Semaglutide further extends this principle through structural modifications that support once-weekly exposure, and a randomized pharmacokinetic study reported terminal half-life values of approximately 145–167 h at steady state [149]. Similarly, tirzepatide, a dual GIP/GLP-1 receptor agonist, is highly albumin-bound and has a half-life of approximately five days, supporting once-weekly administration [150]. These comparisons show that albumin-binding moieties do not simply improve peptide stability in a general sense; they can shift therapeutic feasibility from minute-scale or hour-scale exposure to once-daily or once-weekly pharmacokinetic profiles.

### 6.4. Catalytic Peptide-Bond Formation and Late-Stage Peptide Modification

Catalytic peptide-bond formation is emerging as a more sustainable alternative to conventional coupling-reagent-based peptide synthesis because it can reduce stoichiometric activators, reagent-derived waste, and solvent-intensive purification [151]. A key advance was the development of the 1,3-dioxa-5-aza-2,4,6-triborinane (DATB) catalyst, whose B_3_NO_2_ ring system enables direct dehydrative amidation through cooperative boron-centercentered substrate activation [152]. Further development of this approach showed that catalytic oligopeptide synthesis can be achieved using organoboron catalysis, supporting the feasibility of catalyst-controlled peptide-bond formation beyond simple amide substrates [153]. More recent redox organocatalytic systems have extended this concept by enabling peptide-bond formation through organoreductant/organooxidant recycling, thereby reducing reliance on conventional stoichiometric coupling reagents [154]. A later small-molecule catalyst further advanced this strategy by enabling peptide synthesis in both the solution and solid phase, including compatibility with acetonitrile and reduced dependence on problematic solvents such as DMF (dimethyl formamide) [155]. Recent N(BOH)_2_-based amidation catalysts and organoboron-catalysis reviews further support the rapid development of direct dehydrative amide/peptide-bond formation as a green-chemistry direction for peptide synthesis [151].

In contrast, palladium-catalyzed cross-coupling, copper-catalyzed azide–alkyne cycloaddition, ruthenium-mediated olefin metathesis, photoredox C–H functionalization, and electrochemical disulfide formation are better classified as late-stage peptide modification strategies [156,157]. These reactions are valuable for installing macrocycles, staples, labels, side-chain modifications, or stability-enhancing motifs after peptide assembly, but they should not be presented as primary peptide-bond-forming methods.

## 7. Nanoformulations and Barrier-Crossing Strategies for Peptide Delivery

### 7.1. Overcoming Biological Barriers

To bypass protective physiologic barriers, engineered peptides target specific, barrier-related biochemical pathways for selective entry [158]. Cell-penetrating peptides (CPPs) that include arginine-rich CPPs such as TAT (transactivator of transcription) and penetratin can enter cells by disrupting a portion of their lipophilic membrane directly; through transient disruption of their endoplasmic reticulum/lipid bilayer structure; or through endocytic pathways [159]. Endocytic pathways may be mediated by sequence domains in CPPs, which disrupt the endosome/lysosome fusion process (HA2) (hemagglutinin 2-deriveddomain/peptide), or by domains that bind to receptors on cancer cells (e.g., neuropilin-1) [160]. This helps prevent non-specific toxicity in normal cells and limits the likelihood of degradation within an acidic endolysosomal compartment. In contrast, skin-penetrating peptides (SKPs) such as TD-1 utilize reversible fluidization of the lipid matrix of the stratum corneum and bind to intracellular keratins to allow macromolecules to cross into underlying dermis tissue layers without causing irreversible damage to the stratum corneum [161]. Tight junction-modulating peptides (TJMPs) are used to deliver therapeutic agents systemically to mucosal surfaces. TJMPs such as Zot, derived from the Vibrio cholerae toxin and larazotide, modulate tight junctions between adjacent epithelial cells by competitively inhibiting the interaction between claudin and occluding proteins, or by removing the structural divalent cations required for these protein interactions [162,163].

### 7.2. Self-Assembling Peptide Nanostructures

Amphiphilic peptide molecules assemble into well-defined supramolecular structures. These supra-molecular structures can exhibit different forms of morphology [164]. For example, the Diphenylalanine (FF) sequence repeats will produce very rigid nanotube constructs, which mimic synthetic ion channel conductors having a Young’s modulus near 20 GPa [165]. Alternating self-complementary sequence repeats (i.e., RADA16), as well as Fmoc-modified peptides, will form nano-fibrous networks of beta-sheets, which contain approximately 99.9% water and can be formed into macroporous hydrogel constructs [166]. Importantly, when subjected to flow, the supra-molecular matrices have been found to be shear-thinning materials and therefore can be easily injected through a syringe with minimal invasion and then instantaneously gelled in situ to provide local drug delivery systems with extended duration [95]. The supra-molecular matrices described herein are highly programmable and may act as synthetic ECMs (Extracellular Matrices), providing surface motifs for cell attachment (such as RGD (arginine–glycine–aspartic acid), YIGSR (Tyr-Ile-Gly-Ser-Arg)) to stimulate tissue repair/regeneration or as responsive reservoirs that deconstruct and release entrapped therapeutic agents based on changes in their micro-environmental conditions (such as pH, enzymatic digestion, heat) [167].

Beyond their structural tunability, self-assembling peptide systems are increasingly being developed as functional biomedical platforms for localized drug delivery, tissue-regenerative scaffolding and image-guided biomaterial monitoring. Recent work on multicomponent peptide/iron(III)-based hydrogels showed that a self-assembled peptide matrix can incorporate a stable Fe(III) complex to generate an injectable T_1_ magnetic resonance imaging-visible scaffold, supporting non-invasive monitoring of hydrogel localization and degradation [168]. Bioinspired short-peptide hydrogels demonstrated the importance of sequence and terminal-group design, as pentapeptide systems derived from nucleophosmin 1 showed tunable hydrogelation, fibrillar nanostructures, shear-thinning behavior, and biocompatibility [169]. Naproxen-conjugated self-assembling peptide hydrogels have also been reported for osteoarthritis treatment, where NpxFFK improved joint retention and sustained anti-inflammatory activity, macrophage polarization, and therapeutic outcomes in an osteoarthritis rat model [170]. In tissue repair, RADA16-I-based hydrogels functionalized with elastase-cleavable bioactive motifs improved fibroblast/keratinocyte compatibility and enhanced wound healing in mice. While RADA-PDGF2 hydrogels were designed to provide enzymatically responsive release of a platelet-derived growth factor-BB-derived wound-healing motif [171]. Self-assembling peptide hydrogels have also been explored for local anticancer therapy, as pH-responsive peptide hydrogels loaded with doxorubicin showed tumor-microenvironment-responsive release, injectability, biocompatibility, and antitumor efficacy in mouse models [172]. Importantly, RADA16-based materials also have clinical relevance as topical hemostatic matrices, with a randomized controlled trial showing reduced need for heat therapy during endoscopic submucosal dissection and improved wound healing at four weeks [173]. Collectively, these examples indicate that self-assembling peptide materials should not be interpreted only as supramolecular structures.

### 7.3. Carrier-Based Nanoformulations for Peptide Delivery

Nanoencapsulation of unstable peptides is accomplished using a carrier-based matrix design to enable the desired release characteristics, limit proteolysis, and greatly extend the time these peptides are present at the site of action [174]. Lipid-based delivery systems such as nanostructured lipid carriers (NLCs) and solid lipid nanoparticles (SLNs) contain aqueous domains or lipid bilayer regions where hydrophilic peptide segments can be shielded from opsonins [175]. Additionally, bioabsorbable polymeric nanospheres made from FDA-approved polymers such as PLGA (poly(lactic-co-glycolic acid)), and/or mucoadhesive materials capable of opening tight junctions, such as chitosan, will control the rate of drug release through predetermined diffusion-erosion mechanisms [176]. Even greater versatility has been demonstrated by advanced inorganic systems, which provide additional options for protecting/delivering peptides. For example, mesoporous silica nanoparticles (MSNs) contain large surface area pore volumes where peptides can reside until removed after stimulus-mediated cap removal [177]. Gold nanoparticles (AuNPs) have also been engineered to take advantage of their unique optical properties, as evidenced by surface plasmon resonance, to deliver drugs/photothermally and simultaneously image target sites for therapy [178]. When peptides are combined with natural biopolymer-based scaffolds such as nanocellulose, gelatin, or silk fibroin, they form hybrid composite systems that integrate the structural/mechanical advantages of the biopolymer scaffold with the specific bioactivities of the peptide to develop highly effective infection-resistant wound dressings and implantable/bioelectronic interfaces [95,179].

### 7.4. Targeted Linkers and Conjugates

Peptide–drug conjugates (PDCs) and antibody–drug conjugates (ADCs) most often employ cleavage motifs for Cathepsin, such as the highly stable plasma–valine–citrulline (Val-Cit) motif, linked via an auto-degradative para-aminobenzylcarbamate (PABC) spacer that releases unaltered cytotoxic agents (i.e., MMAE (monomethyl auristatin E) in the FDA-approved Brentuximab vedotin) exclusively after entering the proteolytically rich lysosome compartment [42].

**Figure 3:**
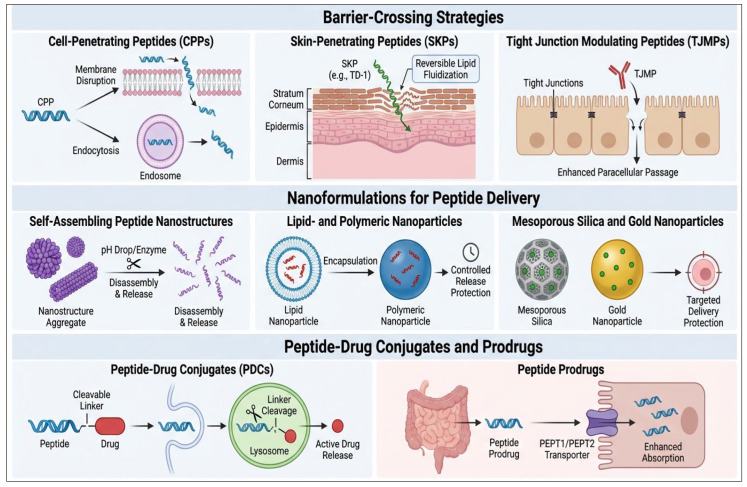
Nanoformulations and barrier-crossing strategies for peptide delivery. This figure illustrates strategies for enhancing peptide delivery through nanoformulations and barrier-crossing techniques. It covers cell-penetrating peptides (CPPs), skin-penetrating peptides (SKPs), and tight junction-modulating peptides (TJMPs), along with self-assembling peptide nanostructures for controlled release. It also highlights lipid- and polymer-based nanoformulations, mesoporous silica nanoparticles (MSNs), gold nanoparticles (AuNPs), and peptide–drug conjugates (PDCs) to improve stability, targeting, and bioavailability. Created and edited in BioRender. Zaman, W. (2026) https://BioRender.com/f66pzgm. Accessed on 20 April 2026.

In addition, peptide prodrug approaches have substantially improved drug pharmacokinetics; drugs can be esterified using amino acids to exploit intestinal transporters PEPT1/PEPT2 (peptide transporter ½) to improve oral bioavailability from 0.5- to 10-fold (example: valacyclovir) [180,181]; furthermore, drugs are masked with phosphates to significantly increase their water solubility due to the elimination of their positive charge [182]. Furthermore, the integration of temporarily shielded architectures with MMP-activatable (matrix metalloproteinase) or light-activatable cleavage sites will allow for payload inactivity throughout systemic circulation and activation in selected tumor or inflammatory environments to provide maximal enhancement of the therapeutic window [183] (Figure 3).

## 8. Applications of Peptides

Clinically established peptide therapeutics, including GLP-1 receptor agonists, selected antimicrobial peptides, ziconotide, and peptide-based conjugate systems, explain the therapeutic value of peptide scaffolds. In contrast, cosmeceutical peptides and peptide-based biosensors should be interpreted as application-adjacent technologies rather than equivalent therapeutic categories. This distinction is important to maintain a clear boundary between approved or clinically advanced peptide medicines and experimental or supportive biomedical applications.

### 8.1. Therapeutic Applications and Topical Peptide Technologies

Cosmeceutical peptides are topical peptide technologies and must not be confused as systemic peptide therapeutics. Their relevance to pharmaceutical peptide science lies in local delivery, skin penetration, receptor or matrix signaling, formulation stability, and safety evaluation rather than in conventional systemic pharmacology. AMPs like Daptomycin disrupt the membranes of bacteria through a variety of methods that significantly delay or reduce the ability of bacteria to develop drug resistance [184]. GLP-1 receptor agonist peptides (such as Semaglutide and Tirzepatide) also have a significant impact on metabolic disease [103]. The combination of increasing the length of time that natural incretin hormones exist in the body, using fatty acid modification of the hormone to increase its stability and extend its half-life, has allowed for greater than ever before glycemic control and potentially 25% or greater weight loss [185]. Peptide-based treatments can also be used in targeted therapy for cancer. By using specific targeting sequences (such as RGD motifs and Chlorotoxin) to deliver cytotoxic agents and radioactive isotopes to cancer cells, researchers have developed highly targeted theranostics [186,187]. The cosmetic industry is also utilizing peptide technology. The cosmeceutical market utilizes various forms of signaling peptides (such as Palmitoyl-Pentapeptide-4) to stimulate collagen production [188]. The cosmeceutical market is also utilizing neurotransmitter-inhibiting peptides such as Argireline (acetyl hexapeptide-8; sequence: Ac-EEMQRR-NH_2_) that inhibit the release of acetylcholine, which mimics botulinum toxin by attenuating facial muscle contractions, thereby reducing the clinical appearance of rhytids [189].

### 8.2. Peptide-Based Diagnostics as Translational Support Platforms

**Figure 4:**
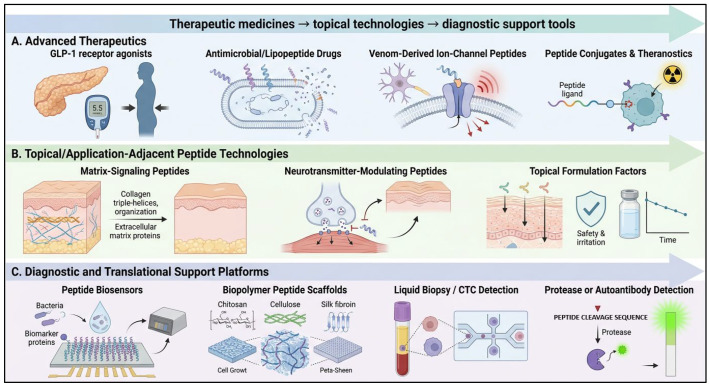
Applications of peptides in therapeutics, cosmeceuticals, and diagnostics. This figure highlights the diverse applications of peptides in therapeutic, cosmeceutical, and diagnostic fields. It covers the use of antimicrobial peptides (AMPs) in drug resistance, GLP-1 receptor agonists for metabolic diseases, targeted peptide therapies in cancer, and peptides in cosmeceuticals for collagen stimulation and wrinkle reduction. Created and edited in BioRender. Zaman, W. (2026) https://BioRender.com/f66pzgm Accessed on 20 April 2026.

Researchers develop “smart” bio-recognition surfaces, capable of detecting femtomolar levels of disease-specific biomarkers by conjugating peptide sequences onto various biopolymer scaffold structures (e.g., chitosan, cellulose nanofibers, hyaluronic acid, silk fibroin) [125,190]. These engineered hybrid systems are compatible with multiple transducer architectures; e.g., an antimicrobial-peptide-based electrochemical sensor made from chitosan will detect bacteria within 30 min, and a peptide-functionalized silk-fibroin scaffold system is able to detect CTCs (circulating tumor cells) for the purpose of liquid biopsy [190,191]. The primary design focus of both types of systems differs significantly from those used to deliver drugs via encapsulation and controlled release. Rather than focusing on encapsulating drugs for slow-release into a body compartment or organ, the primary goal is to provide a high-density, high-activity surface functionality for maximum target interaction [95]. Therefore, researchers can rapidly and concurrently detect autoantibodies and cancer-related proteases directly at the point-of-care [192] (Figure 4, Table 3).

## 9. Current Challenges and Emerging Research Gaps

Although there has been tremendous growth in the development of peptide drugs as therapeutics, many key issues have still limited their broader use in a clinical setting. First, from a synthetic standpoint, chemically sustainable “green” synthesis techniques for peptide segments containing sulfur linkages, which are extremely sensitive to degradation, and ways to stabilize such sequences, remain underutilized [199]. Second, the long-term immune response and cost impact associated with the higher levels of PEGylation used in advanced formulations will likely require additional long-term studies [200,201]. Third, in terms of delivery architectures, including both CPPs and self-assembling nano-structures, there exist significant barriers to translation due to poorly defined formation mechanisms, the lack of theoretical predictive models, and no FDA-approved CPP formulation [202]. Fourth, although the utilization of naturally occurring peptides (from marine, insect, venom, and food waste) is potentially rich with therapeutic opportunities, it remains constrained by non-standardized extraction procedures; variability in yield; and an over-reliance on in vitro data that does not confirm in vivo bioavailability/toxicity/allergenicity [203,204]. Finally, achieving consistent peptide immobilization for smart biosensing devices and maintaining an appropriate balance between linker stability in conjugates also represent significant challenges [205], indicating the need to utilize artificial intelligence for de novo optimized sequencing, structural prediction, and targeted resolution of the above-noted translational roadblocks [206] (Table 4).

## 10. Conclusions

The development trajectory of peptide therapeutics has transformed from the accidental identification of unstable natural hormone peptides to the design and construction of stable, multi-functional engineered proteins. A new generation of peptide drugs was made possible by advances in three key areas: (i) artificial intelligence (AI)-driven in silico drug discovery; (ii) environmentally friendly (green-chemistry) methods for scaling-up synthetic procedures; and (iii) advanced structural modification techniques designed to increase the stability of peptides (hydrocarbon stapling; backbone N-methylation; and conjugation of peptides). As a result, peptides can now readily overcome previously recognized barriers to use, including poor pharmacokinetics. This transformation in the design and application of peptide drugs has been verified in multiple therapeutic fields: long-lasting GLP-1 receptor agonist therapies are transforming our treatment strategies for diseases such as type II diabetes; antimicrobial peptides (AMPs) represent an emerging class of antibiotics which can address the rising threat of antibiotic resistance; peptide–drug conjugate (PDC) therapies are creating new paradigms for cancer therapy based on targeted molecular recognition; and bioactive peptide sequences are providing a scientific basis for the evaluation of efficacy and safety in advanced cosmeceutical products. With over 100 approved peptide therapeutics currently marketed, along with a strong pipeline of greater than 550 additional peptide therapeutics in various stages of clinical or preclinical testing, peptides may soon match the efficacy of small molecule drugs and monoclonal antibody treatments in terms of their overall impact on health outcomes.

In addition to its current rapid growth as a field, the future growth of therapeutic peptide research will be further accelerated by the transition of generative AI algorithms from secondary screening tools to primary de novo sequence design platforms. These new platform technologies will predict with a high degree of accuracy multi-dimensional ADMET characteristics associated with potential therapeutics. In order to reduce the historically significant barrier to the use of orally administered peptide therapeutics due to poor absorption, researchers are using combinations of retro–inverso scaffold designs, tight junction modulators, and nanoparticle encapsulation to improve oral bioavailability. The next phase of this rapidly evolving field includes conditionally activated theranostic agents, multi-targeted agonists, and hybrids that combine peptide carriers with CRISPR-Cas9-mediated gene editing and/or regenerative tissue repair. Although there remain several significant obstacles to be addressed before these peptide therapeutics become commercially viable, it is clear that they have already exceeded their natural limitations and have established themselves as the most versatile scaffolding systems available today for designing novel precision medicines and sustainable pharmaceuticals.

## Figures and Tables

**Table 1:** Natural sources, representative peptides and translational features of therapeutic peptides.

Source	Major Peptide Types	Common Peptide Examples	Key Activities	Main Limitation	Ref
Plants	Seed, cereal, and defense peptides	Lunasin, Bowman–Birk inhibitor, Soy peptides (Glycinin fragments)	ACE inhibition, antioxidant, anti-diabetic	Variable yield, low bioavailability	[51]
Animals	Meat, dairy, collagen, fish peptides	Val-Pro-Pro (VPP), Ile-Pro-Pro (IPP), Casomorphins, Collagen peptides	Antihypertensive, antioxidant, opioid-like	Poor stability, oral delivery barriers	[52]
Microbes	RiPPs, NRPS peptides, cyclic lipopeptides	Nisin, Surfactin, Iturin, Cyclosporine	Antimicrobial, immunosuppressive, anticancer	Toxicity, production scalability	[53]
Algae/Marine	Seaweed, algae, sponge-derived peptides	Didemnins, Dolastatins, Fucoidan-associated peptides	ACE inhibition, cytotoxic, antioxidant	Complex purification, safety concerns	[54]
Venoms	Disulfide-rich ion-channel peptides	Chlorotoxin, ω-Conotoxin MVIIA (Ziconotide), Melittin, Disintegrins	Analgesic, anticancer, antimicrobial	Toxicity, immunogenicity	[55]

The amino acid sequences of representative peptide drugs discussed in this review are summarized in Table S1.

**Table 2:** Structural taxonomy and pharmacological profiles of therapeutic peptides.

Structural Class	Defining Feature	Key Pharmacological Advantage	Representative Examples	Ref
Linear peptides	Flexible, unbranched amino acid chains	Simple synthesis; adaptable target binding but low stability	Carnosine, glucagon, GLP-1	[96]
Cyclic peptides	Covalently closed peptide backbone	Enhanced stability, affinity, and resistance to proteolysis	Cyclosporine, polymyxins	[97]
Disulfide-rich peptides	Intramolecular S–S bonds forming constrained 3D structures	High structural rigidity and receptor specificity	Oxytocin, defensins, conotoxins	[98]
Stapled peptides	Chemically constrained α-helices via hydrocarbon staples	Enhanced helicity, protease resistance, and intracellular delivery	ALRN-6924	[99]
Modified peptides/peptidomimetics	Incorporation of non-natural residues or backbone changes	Improved metabolic stability and membrane permeability	D-peptides, peptoids	[98]
Lipidated/glycosylated peptides	Conjugation with fatty acids or glycans	Extended half-life, improved solubility and PK	Liraglutide, semaglutide	[96]
PEGylated peptides	Covalent attachment of PEG polymers	Reduced renal clearance and immunogenicity; prolonged circulation	Peginterferon alfa	[100]
Peptide–drug conjugates/prodrugs	Peptide linked to cytotoxic or functional payload	Targeted delivery and improved therapeutic index	Brentuximab vedotin, valacyclovir	[101]
Self-assembling peptides	Non-covalent supramolecular assembly (π–π, hydrophobic interactions)	Formation of nanostructures for delivery and biomaterials	Diphenylalanine nanotubes, Fmoc-peptides	[102]

**Table 3:** Comparative translational status of peptide technologies discussed in this review.

Peptide Technology	Examples	Current Status	Pharmaceutical Value	Key Limitation	Ref
Peptide hormones/incretin analogs	Insulin, liraglutide, semaglutide, tirzepatide	Approved/clinically established	Long-acting metabolic therapy	Injection burden, cost, adverse effects	[13]
Antimicrobial/lipopeptide drugs	Daptomycin, polymyxins, topical AMPs	Approved for selected agents	Anti-infective therapy	Toxicity, resistance, narrow window	[193]
Venom-derived ion-channel peptides	Ziconotide/ω-conotoxin MVIIA	Approved for refractory pain	Selective ion-channel targeting	Intrathecal delivery, neurotoxicity risk	[194]
Peptide receptor radionuclide therapy/conjugates	^177^Lu-DOTATATE, PDCs	Approved for PRRT; PDCs emerging	Targeted payload delivery	Off-target uptake, linker stability	[195]
Stapled/constrained peptides	ALRN-6924, hydrocarbon-stapled peptides	Clinical/preclinical	Intracellular target engagement	Delivery and exposure limits	[137]
Self-assembling peptide biomaterials	RADA16, PuraStat, peptide hydrogels	Clinical for hemostasis; many preclinical	Local delivery, hemostasis, scaffolds	Reproducibility, degradation control	[173]
Cell-penetrating/barrier-crossing peptides	TAT, penetratin, SKPs, TJMPs	Mostly experimental	Intracellular and mucosal delivery	Poor selectivity, clearance, toxicity	[196]
AI-designed peptides	AMPGAN, APEX 1.1, ProteoGPT/AMPGenix	Preclinical/rapidly developing	Faster sequence discovery	Dataset bias, weak PK prediction	[89]
Cosmeceutical topical peptides	Palmitoyl-pentapeptide-4, Argireline	Commercial/topical	Skin delivery and matrix signaling	Variable evidence and regulation	[197]
Peptide biosensors/diagnostics	Peptide-functionalized sensors, protease probes	Translational support/proof-of-concept	Biomarker detection and monitoring	Clinical validation, surface stability	[198]

**Table 4:** Key challenges and research gaps in pharmaceutical peptides.

Domain	Core Challenge	Impact	Research Direction	Ref
Green synthesis	Poor sustainability for sulfur-rich peptides	Limits scalable production	Develop mild, low-waste synthesis	[207]
PEGylation	Uncertain long-term safety and cost	Limits chronic use	Biodegradable/cleavable polymers	[208]
CPP delivery	Poor selectivity, unclear uptake	Limits clinical translation	Targeted, stimuli-responsive CPPs	[209]
Self-assembly	Unpredictable structure and stability	Regulatory and reproducibility issues	AI/MD-guided design	[210]
Natural peptides	Variable extraction and weak in vivo data	Poor translation beyond screening	Standardized pipelines, in vivo validation	[211]
Conjugates	Linker instability vs. release control	Reduced efficacy/safety	Stimuli-responsive linkers	[212]
Immobilization	Poor surface stability/orientation	Low biosensor reliability	Site-specific conjugation	[213]
AI design	Limited predictive accuracy (PK/ADME)	Low translation success	Integrated AI + experimental validation	[98]

## Data Availability

No new data were created or analyzed in this study.

## Supplementary Materials

The following supporting information can be downloaded at: https://www.mdpi.com/article/10.3390/ph19060811/s1, Table S1: Amino acid sequences or structural notations of representative peptide drugs.
