# Supplementary material for: Pharmaceutical Peptides: From Synthesis and Mechanistic Pharmacology to Future Biologic Therapeutics
Source: Pharmaceuticals (Basel). 2026 May 22;19(6):811. doi: 10.3390/ph19060811 (PMC13304512; doi:10.3390/ph19060811)
Supplement: Supplementary file 1 [file pharmaceuticals-19-00811-s001.zip › pharmaceuticals-4312065-supplementary.pdf]

pharmaceuticals

Review

Pharmaceutical Peptides: From Synthesis and Mechanistic Pharmacology to Future Biologic Therapeutics

Muhammad Yaseen Khan [1], Touseef Nawaz [1], Muhammad Sajid Hamid Akash [2] and Adnan Amin [3,*]

[1] Department of Pharmacy, Qurtuba University of Science and Information Technology, Peshawar 25100, Pakistan
[2] Department of Pharmaceutical Chemistry, Government College University, Faisalabad 54000, Pakistan
[3] Department of Life Sciences, Yeungnam University, Gyeongsan 38541, Republic of Korea
* Correspondence: adnan.amin@yu.ac.kr

 https://doi.org/10.3390/ph19060811

Table S1. Amino acid sequences or structural notations of representative peptide drugs

**Peptide drug/example**	**Amino acid sequence / structural notation**	**Notes**
Human insulin	A-chain: GIVEQCCTSICSLYQLENYCN; B-chain: FVNQHLCGSHLVEALYLVCGERGFFYTPKT	Two-chain peptide hormone linked by disulfide bonds
Val-Pro-Pro	VPP	Casein-derived antihypertensive tripeptide
Ile-Pro-Pro	IPP	Casein-derived antihypertensive tripeptide
Ziconotide / ω-conotoxin MVIIA	CKGKGAKCSRLMYDCCTGSCRSGKC-NH_2	C-terminally amidated peptide with three disulfide bridges
Chlorotoxin	MCMPCFTTDHQMARK-CDDCCGGKGRGKCYGPQCLCR-NH_2	36-amino-acid scorpion venom peptide; disulfide-rich
Melittin	GIGAVLKVLTTGLPALISWIKRKRQQ-NH_2	Amphipathic bee-venom peptide
Eptifibatide	cyclo[Mpa-Har-Gly-Asp-Trp-Pro-Cys]-NH_2	Cyclic heptapeptide; Mpa = mercaptopropionyl, Har = homoarginine
Cyclosporine A	cyclo[MeBmt-Abu-Sar-MeLeu-Val-MeLeu-Ala-D-Ala-MeLeu-MeLeu-MeVal]	Cyclic undecapeptide; use modified-residue notation
Nisin	Modified 34-residue lantibiotic containing dehydrated residues and lanthionine/methyllanthionine bridges	Do not present as a simple unmodified linear sequence
Daptomycin	N-decanoyl-Trp-D-Asn-Asp-Thr-Gly-Orn-Asp-D-Ala-Asp-Gly-D-Ser-3-MeGlu-Kyn	Cyclic lipopeptide; contains D-amino acids and noncanonical residues
GLP-1(7–36) amide	HAEGTFTSDVSSYLEGQAAKEFIAWLVKGR-NH_2	Native active GLP-1 fragment
Liraglutide	HAEGTFTSDVSSYLEGQAAK[γ-Glu-palmitoyl]EFI-AWLVRGRG	Lipidated GLP-1 analogue; C16 fatty-acid modification
Semaglutide	H-Aib-EGTFTSDVSSYLEGQAAK[AEEA-AEEA-γ-Glu-C18 diacid]EFIAWLVRGRG-OH	GLP-1 analogue with Aib substitution and C18 fatty-diacid linker
Tirzepatide	YXEGTFTSDYSIXLDKIAQKAFVQW-LIAGGPSSGAPPPS-NH_2, where X = Aib; Lys20 carries a C20 fatty-diacid linker	Dual GIP/GLP-1 receptor agonist; 39-amino-acid lipidated peptide
Palmitoyl-pentapeptide-4	Pal-KTTKS or Palmitoyl-Lys-Thr-Thr-Lys-Ser	Cosmetic signaling peptide
Argireline / acetyl hexapeptide-8	Ac-EEMQRR-NH_2	Synthetic hexapeptide used in cosmeceuticals

https://doi.org/10.3390/ph19060811
